# Distinct Patterns of University Students Study Crafting and the Relationships to Exhaustion, Well-Being, and Engagement

**DOI:** 10.3389/fpsyg.2022.895930

**Published:** 2022-06-10

**Authors:** Lina Marie Mülder, Sonja Schimek, Antonia Maria Werner, Jennifer L. Reichel, Sebastian Heller, Ana Nanette Tibubos, Markus Schäfer, Pavel Dietz, Stephan Letzel, Manfred E. Beutel, Birgit Stark, Perikles Simon, Thomas Rigotti

**Affiliations:** ^1^Department of Work, Organizational and Business Psychology, Institute of Psychology, Johannes Gutenberg University Mainz, Mainz, Germany; ^2^Department of Psychosomatic Medicine and Psychotherapy, University Medical Centre, Johannes Gutenberg University Mainz, Mainz, Germany; ^3^Institute of Occupational, Social and Environmental Medicine, University Medical Centre of the University of Mainz, Mainz, Germany; ^4^Department of Diagnostics in Healthcare and E-Health, University of Trier, Trier, Germany; ^5^Department of Communication, Johannes Gutenberg University Mainz, Mainz, Germany; ^6^Department Sport Medicine, Rehabilitation and Disease Prevention, Institute of Sport Science, Johannes Gutenberg University Mainz, Mainz, Germany; ^7^Leibniz Institute for Resilience Research, Mainz, Germany

**Keywords:** university students, well-being, burnout, study crafting, health promotion

## Abstract

Job crafting has been established as a bottom-up work design instrument for promoting health and well-being in the workplace. In recent years, the concepts of job crafting have been applied to the university student context, proving to be positively related to student well-being. Building on person-centered analyses from the employment context, we assessed approach study crafting strategy combinations and the relationships to students’ exhaustion, study engagement, and general well-being. Data from 2,882 German university students were examined, collected online during the summer term in 2020. Using latent profile analysis, we found five distinct crafting groups, which showed discriminate validity with regard to emotional exhaustion, engagement, and well-being. The results underscore the positive role of study crafting for students’ health and well-being. They further indicate a less important role of increasing social resources for emotional exhaustion when combined with a moderate increase in structural resources and a moderate increase in challenging demands. Our findings imply that interventions to promote study crafting should be considered to promote student health and well-being.

## Introduction

International studies show a high prevalence of depression, anxiety, and other mental disorders among university students (Bayram and Bilgel, 2008; Shamsuddin et al., 2013; Auerbach et al., 2016), and these numbers have increased since the COVID-19 outbreak (Li et al., 2021; Werner et al., 2021). Many students had fewer opportunities during the pandemic to celebrate their milestones, such as graduations or exams, and also had to cancel or indefinitely postpone internships or study abroad programs (Lederer et al., 2021). For many students, attending university represents a challenging period in their lives (Wing et al., 2018; Kaggwa et al., 2021). University students face content-related cognitive demands from their studies, such as exams and lectures. They need to structure their daily routines in response to new demands in their private environment and cope with developmental challenges concerning their identity formation. As a result, students may feel overwhelmed and emotionally exhausted (Kriener et al., 2018). Academic burnout is a multidimensional syndrome, including being exhausted from studying, cynicism, and reduced efficacy (Schaufeli et al., 2002a). In the study demands-resources framework (Lesener et al., 2020), high study demands are proposed to be associated with higher burnout risk. Burnout symptoms are known to have negative effects on academic performance (Wing et al., 2018; Madigan and Curran, 2021). Given the high prevalence of burnout and psychological disorders among students, there is an urgent need to learn more about evidence-based approaches for promoting health in higher education. The health of university students is of great societal interest since students will later pursue leadership positions, and their health status and behaviour will likely influence others (Dietz et al., 2020).

Academic study conditions are comparable to job demands and job resources (Clements and Kamau, 2018; Lesener et al., 2020). The pandemic has dramatically pushed trends towards a more digitized learning environment for university students. Digital teaching formats not only give more autonomy to students, but also increase demands on time and self-management (Hoss et al., 2021). Students have a high responsibility for shaping their learning conditions. Adapted from the concept of job crafting used in occupational contexts (Wrzesniewski and Dutton, 2001; Tims et al., 2012), study crafting refers to the changes that students can make to adapt their study demands and resources to suit their personal skills and needs (cf., Dormann and Guthier, 2019; Körner et al., 2021). Tims et al. (2012) proposed four dimensions of job crafting: (1) increasing structural resources, (2) increasing social resources, (3) increasing challenging demands, and (4) decreasing hindering demands. We adapted the same differentiation for study crafting. These distinct crafting dimensions can be grouped into approach and avoidance crafting (Mäkikangas, 2018; Zhang and Parker, 2019; Lopper et al., 2020). Meta-analyses reported a positive relationship of avoidance crafting (i.e. decreasing hindering demands) with burnout (Rudolph et al., 2017; Lichtenthaler and Fischbach, 2019), and either non-significant (Lichtenthaler and Fischbach, 2019) or even a negative relationship to work engagement (Rudolph et al., 2017). The health-promoting effect of approach job crafting (increasing structural, social resources, and challenging demands) is undisputed, and training courses have been designed to promote employee job crafting (Oprea et al., 2019; Demerouti et al., 2021). Our study aims to contribute to recent research on the adaptation of job crafting to the university context (Dormann and Guthier, 2019; Ferreira, 2020; Körner et al., 2021). The different crafting strategies can be employed in different constellations. We built upon a study by Mäkikangas (2018), who used a latent cluster approach in a sample of employees and found two clusters, differentiating between active and passive job crafters. Active job crafters mainly used approach crafting techniques, whereas passive job crafters reported more avoidance crafting. In contrast to Mäkikangas (2018), we will only focus on approach crafting dimensions in our analyses, seeking a more differentiated typology within this category. As avoidance or passive crafting is mainly based on the strategy to decrease hindering demands, no further differentiation for this crafting type can be expected, at least with the concept by Tims et al. (2012). Therefore we focus on approach crafting strategies. We will use latent profile analyses (LPA) to classify participants into homogeneous subgroups, sharing a similar combination of using different crafting strategies. These different configurational profiles can be characterized by corresponding personal or conditional aspects (Spurk et al., 2020). Compared to the variable-centred approach, a person-centered approach allows for a more holistic picture concerning the interplay of different crafting strategies.

By employing a person-centered approach in a large sample of university students, we hope to find a further differentiation of distinct combinations of approach crafting strategies. Differences between these profile types will be investigated with regard to exhaustion, as the leading symptom of study burnout, study engagement, as a positive motivational, emotional state, as well as general well-being. The aims of our study are to provide further evidence for the applicability of the crafting concept in the higher education context, as well as to discuss evidence-based implications for the development of study crafting interventions as one promising pathway for health-promotion among university students.

### Job and Study Crafting

The concept of job crafting can be defined as ‘self-initiated change behaviours that employees engage in with the aim to align their jobs with their own preferences, motives, and passions (Wrzesniewski and Dutton, 2001; see also Berg et al., 2010)’ (Tims and Bakker, 2010, p. 173). There are different conceptualizations of job crafting. We will focus here on the conceptualization based on the job demands-resources model (JD-R; Bakker and Demerouti, 2017), differentiating between increasing structural resources, increasing social resources, increasing challenging demands, and decreasing hindering demands (Tims and Bakker, 2010; Tims et al., 2012).

The JD-R was adapted to the higher education context (Clements and Kamau, 2018; Lesener et al., 2020). Students cope with comparable demands like time-, and performance pressure and access similar resources like support and autonomy as employees. Likewise, the concept of job crafting has been applied to the study context (Ferreira, 2020; Körner et al., 2021). Increasing structural study resources can include, for example, asking lecturers for more latitude; increasing social study resources can be achieved by proactively asking lecturers for feedback; and increasing challenging study demands is about getting involved in new and interesting projects. On the other hand, reducing hindering study demands corresponds to postponing or avoiding too mentally or emotionally demanding tasks. In a study among first-year students in South Africa, an increase in structural resources was positively related to study engagement (Ferreira, 2020), but an increase in social resources was not associated with increased engagement in this sample. In a weekly diary study in a sample of higher education students enrolled at a German university, study resources were shown to be indirectly related to an increase in structural and social resources *via* increased study engagement (Körner et al., 2021).

Data collected by Mäkikangas (2018) from Finnish rehabilitation center employees using latent cluster analysis revealed two distinct profiles of job crafting behaviour. The vast majority of employees (94%) representing the actively crafting group showed slightly more crafting in all dimensions. The second group comprised the so-called passive job crafters (6%), who mainly engaged in reducing hindering demands. The group that showed more approach crafting behaviour scored higher in work engagement. Similar findings on the distinctions between the two domains of approach (promotion) and avoidance (prevention) have been reported in meta-analyses (Rudolph et al., 2017; Bruning and Campion, 2018; Lichtenthaler and Fischbach, 2019; Lazazzara et al., 2020). There were also divergent empirical results related to job crafting dimensions, as proposed by Tims and Bakker (2010). Whereas positive outcomes were reported for increasing structural and social resources, as well as for increasing challenging demands, reducing hindering demands was shown to be negatively related to engagement and satisfaction and positively related to emotional exhaustion (Lichtenthaler and Fischbach, 2019; Zhang and Parker, 2019).

The COVID-19 pandemic changed the way of studying in universities in various ways. The spatial conditions shifted from the university to home, making building and cultivating social support or friendships more complex. Asynchronous teaching formats, delivered by videos and task assignments on demand, resulted in the increased need for self-management (Hoss et al., 2021), and also might have increased opportunities for students to proactively seek more structural resources, and challenging demands.

Our study focused on approach crafting, seeking further differentiation within this category. Two aspects guided our decision: (1) based on the findings reported by Mäkikangas (2018), only a marginal share of respondents (6%) were categorized as avoidance crafters, (2) meta-analytic findings (Rudolph et al., 2017; Lichtenthaler and Fischbach, 2019) showed that avoidance crafting could not be seen as a beneficial strategy (albeit causality for the negative relationship of avoidance crafting with burnout is still an open question). Approach crafting showed consistently positive relationships with motivation, and well-being, and hence should be the focus of interventions aiming at increasing the use of crafting strategies.

To capture the holistic interplay between the different facets of study crafting, we looked for profiles used as potential predictors for different health and motivational outcome criteria. Students may pursue other crafting dimensions more or less compared to employees, and combinations of crafting strategies might well show differential effects for students. In the first step, we looked for homogeneous subsamples of students that could be characterized by similar combinations of study crafting strategies. As LPA is an explorative endeavour, we formulated an open research question:

*R1*: How many study crafting profiles can be distinguished based on the study crafting strategies of approach crafting (increasing structural resources, increasing social resources, and increasing challenging demands), and how are the profiles distributed among students?

### Study Crafting Profiles: Differences in Health and Well-Being

In a second step, we aim to investigate potential differences between the study crafting profiles concerning health and well-being indicators. Building on previous research on job crafting and the JD-R (Bakker and Demerouti, 2017), we used emotional exhaustion and study engagement as outcome variables. Since student health is of overarching importance, we furthermore included general well-being. Whereas emotional exhaustion and engagement are rather domain-specific aspects, general well-being represents an extension to other life domains.

#### Emotional Exhaustion

Emotional exhaustion is described as a chronic state of physical and emotional depletion caused by excessive job demands and continuous hassles (Shirom, 1989; Zohar, 1997). It is characterized by feelings of emotional overextension and exhaustion (Wright and Cropanzano, 1998). Accordingly, emotional exhaustion among students refers to feeling exhausted from study demands (Schaufeli et al., 2002a). A recent review (Rosales-Ricardo et al., 2021) pointed out that prevalence rates for burnout among university students, regardless of their field of study, are high, with an estimated prevalence of 55.4% for the core dimension emotional exhaustion. A recent study by Salmela-Aro et al. (2022) reported increased study burnout over the course of the pandemic from 2020 to 2021.

Increasing structural and social resources, as well as challenging demands, as facets of approach study crafting aim at improving the person-environment fit. Resource gain is proposed to be negatively related to strain within the conservation of resources theory (COR; Hobfoll, 1989). Therefore, we expect that students showing more of these behaviours would report less emotional exhaustion. Meta-analyses (Rudolph et al., 2017) showed that increasing structural resources and challenging demands are associated with less job strain. However, increasing social resources did not show a significant effect in terms of strain. In contrast, another meta-analysis (Lichtenthaler and Fischbach, 2019) concluded that the three dimensions of operationalization of Tims and Bakker (2010) consistently led to less burnout over time. We assume that students who show more approach crafting report less emotional exhaustion based on these findings.

*H1a*: Study crafting profiles are differentially related to emotional exhaustion. Groups of students with consistently high levels of increasing structural, and social study resources and increasing challenging study demands show less emotional exhaustion compared to groups of students which report less crafting behaviour.

#### Engagement

Work engagement is a positive, fulfilling state of mind represented by vigour, dedication, and absorption (Schaufeli et al., 2002b) and was adopted to the university context. Student engagement has shown to be an antecedent for academic performance and is strongly linked negatively to burnout (Salanova et al., 2010). In a weekly diary study, Bakker et al. (2015) reported a link between engagement and psychological resources, positively predicting learning activities during educational group meetings (within-person level). According to the COR theory (Hobfoll, 1989), people who develop surpluses of increasing structural and social resources, as well as challenging demands are likely to experience positive well-being. Since study crafting addresses increasing resources, we supposed that students using more of these strategies would report more engagement. Meta-analyses (Rudolph et al., 2017; Lichtenthaler and Fischbach, 2019; Frederick and VanderWeele, 2020; Hu et al., 2020) provided mixed evidence regarding engagement. The results showed that the dimensions of increasing structural resources, increasing social resources, and increasing challenging demands were positively associated with engagement. However, differences in effect sizes were observed. According to Rudolph et al. (2017), increasing structural resources showed the largest effects in relation to engagement, whereas increasing social resources and challenging demands showed smaller effects. A similar picture emerged in a meta-analysis with a longitudinal perspective (Frederick and VanderWeele, 2020). Here, it became clear that structural resources had a larger effect on employee engagement, and increasing social resources explained less variance, whilst challenging demands showed almost no effect. Yet, another meta-analysis by Hu et al. (2020) reported that increasing social resources are most strongly associated with vigour, dedication, and absorption at the second time point, followed by increasing challenging demands and increasing structural resources with small effects. Lichtenthaler and Fischbach (2019) did not differentiate further here, indicating medium to large effects of all three dimensions with engagement.

Applying these results to the study context was difficult due to the varying sizes of the effects on the different dimensions. Thus, we generally assume that all three study crafting dimensions positively relate to engagement, especially if combined.

*H1b*: Study crafting profiles are differentially related to engagement. Groups of students who consistently report high levels in all dimensions show higher engagement compared to groups of students showing less crafting behaviour.

#### Well-Being

Well-being has been associated with a ‘good life’ or ‘happiness’ (Duy and Yıldız, 2019). A distinction can be made between objective and subjective well-being. We focus on subjective and self-rated well-being. According to the World Health Organization (2012), subjective well-being, is defined as a positive individual experience of life, grounded in a comparison of life circumstances with social norms and values. A person-centered study (Freire et al., 2016) indicates that university students reporting high levels of well-being tend to report higher planning, positive reappraisal, and support-seeking coping strategies to deal with academic stress. Research also emphasizes the importance of well-being by indicating its relation to performance, state anxiety, and empathy (Cotton et al., 2002; Freire et al., 2016). Building on the COR theory (Hobfoll, 1989) and the role of resource gain, we propose that students who increase resources through study crafting report more well-being. A meta-analysis also examined the less studied factor of well-being at work and job crafting (Boehnlein and Baum, 2020). The largest effects were reported for increasing structural resources, followed by medium effects for increasing challenging demands and increasing social resources.

Based on the meta-analytic findings, the combination of increasing structural resources and increasing challenging demands were particularly associated with well-being. We expect similar positive effects of study crafting on well-being in the higher education context. Hence, we propose that the combination of high levels on all study crafting dimensions to be associated with higher well-being.

*H1c*: Study crafting profiles are differentially related to well-being. Groups of students with consistently high levels in all dimensions show better well-being than groups of students reporting less crafting behaviour. The combination of increasing structural resources and increasing challenging demands is especially positively related to well-being.

## Materials and Methods

### Data Collection and Study Design

To recruit participants, we contacted all about 31,000 students of the Johannes Gutenberg-University of Mainz, Germany, by e-mail *via* a central mailing list. The questionnaire was made available online in June 2020. Except for a few courses, teaching was delivered online in a mixture of asynchronous (‘on demand’), and synchronous online courses. Students voluntarily participated in the survey and were informed about the study’s objectives. As an incentive, six vouchers for 20 euros were raffled. The students could choose between a regional help initiative for gastronomy named ‘Mainz help’ or an amazon voucher. The study was performed following the Ethical Principles and Guidelines for the Protection of Human Subjects of Research by the American Psychological Association (APA). Ethical approval of the study was obtained (2020-JGU-psychEK-S008).

### Sample

In total, 3,066 university students answered the online questionnaire. After excluding missing data, a final sample of 2,882 students could be retained. More female than male students responded, with a few participants classifying themselves as diverse. On average, participants were 23.4 (SD = 4.4) years old. The majority of participants were enrolled in a bachelor’s degree program, about one quarter were enrolled in a master’s degree program, and about one quarter studied in programs requiring a state examination. Only very few students were enrolled in expiring qualification programs, such as magister and diploma. The focus of the students’ study ranged across disciplines, including natural and social sciences, as well as humanities, theology, medicine, music, language, and education. Table 1 provides more details of the study sample.

**Table 1:** Description of the study sample.

	*M* (SD)	*N* (%)
Age	23.4 (4.4)	
Semester	4.0 (2.7)	
*Gender*		
Women		2,225 (72.6)
Men		821 (26.8)
Diverse		20 (0.7)
*Field of study*		
STEM^a^		506 (16.5)
Social sciences		493 (16.1)
Humanities		630 (20.5)
Medicine		341 (11.1)
Law and economics		479 (15.6)
Teaching		510 (16.6)
Other		53 (1.7)
*Degree intended*		
Bachelor		1,709 (55.8)
Master		645 (21.0)
State examination		608 (21.6)
Others		22 (0.8)
*Relationship status*		
Single		1,349 (47.0)
In a relationship		1,520 (53.0)
*Living Situation*		
With parents or relatives		1,065 (37.1)
In a student dormitory		301 (10.5)
Alone in an apartment		325 (11.3)
With (spouse) partner and/or child(ren) in one apartment		580 (20.2)
In a shared apartment		600 (20.9)

a Science, Technology, Engineering, and Mathematics.

### Measures

#### Study Crafting

To assess study crafting, we used an abbreviated and adapted version of the German job crafting scale (Lichtenthaler and Fischbach, 2016) based on the job crafting scale of Tims et al. (2012; cf., Körner et al., 2021). The scale was shortened to nine items based on the highest loading items per dimension. Participants indicated their answers on a 5-point Likert scale ranging from 1 (*not true at all*) to 5 (*totally true*). A sample item for *increasing structural resources* reads ‘In my studies, I try to learn new things’ (*α* = 0.84, *N* = 2,810). We measured *increasing social resources* with three items (e.g. ‘I look to my lecturer for inspiration’; *α* = 0.71, *N* = 2,802). For the third dimension of *increasing challenging demands*, we assessed three items (e.g. ‘When an interesting project is to be worked on, I take the initiative and apply myself as a student’; *α* = 0.70, *N* = 2,788). The modified items are provided in Table 2.

**Table 2:** Study crafting items.

No.	Item	English translation (not validated)
	*Increasing structural resources*	
1	Ich versuche meine Fähigkeiten weiter zu entwickeln.^a^	I try to develop my capabilities.^b^
2	Ich versuche, mich selbst professionell weiter zu entwickeln.^a^	I try to develop myself professionally.^b^
3	Im Studium versuche ich, neue Dinge (neben den verpflichtenden ETCs) zu lernen.	In my studies, I try to learn new things (in addition to the obligatory ETCs).
	*Increasing social resources*	
4	Ich bitte meine Dozierenden mich zu coachen.	I ask my lecturer to coach me.
5	Ich frage meine Dozierenden, ob er/sie mit meiner Leistung zufrieden ist.	I ask whether my lecturer is satisfied with my work.
6	Ich schaue auf meine Dozierenden, um Inspiration zu erhalten.	I look to my lecturer for inspiration.
	*Increasing challenging demands*	
7	Wenn ein interessantes Projekt bearbeitet werden soll, ergreife ich die Initiative und bewerbe mich als Studierende.	When an interesting project comes along, I offer myself proactively as a student.
8	Wenn es neue Entwicklungen gibt, bin ich eine/r der Ersten, der diese kennt und ausprobiert.^a^	If there are new developments, I am one of the first to learn about them and try them out.^b^
9	Wenn die Studienbelastung gering ist, sehe ich das als Möglichkeit neue Projekte zu beginnen.	When the study load is low, I see it as chance to start new projects.

a Items are initially from translation of Lichtenthaler and Fischbach (2016) of the job crafting scale (Tims et al., 2012) and needed no adaption to the university student context.

b Items are originally from the job crafting scale (Tims et al., 2012) and did not need to be adapted to the student context.

#### Emotional Exhaustion

To measure *emotional exhaustion*, we used the German Maslach Burnout Inventory for university students (Gumz et al., 2013) based on the Maslach Burnout Inventory student survey of Schaufeli et al. (2002a). We used five items on a 7-point Likert scale ranging from 1 (*never*) to 7 (*daily*). An example item is ‘I feel exhausted from my studies’ (*α* = 0.92, *N* = 2,874).

#### Engagement

To assess *engagement*, we used the ultra-short measure for work engagement (Schaufeli et al., 2017), translated into German by Grützmacher et al. (2018), and adapted the items to the study context. The three items were rated on a 6-point Likert scale ranging from 1 (*never*) to 6 (*always*). An example item is ‘My studies inspire me’ (*α* = 0.84, *N* = 2,813).

#### Well-Being

To measure well-being, we used the German WHO’s Five Well-being Index (Brähler et al., 2007), with five items on a 6-point Likert scale ranging from 0 (*at no time*) to 5 (*all the time*). An example item is ‘The last 2 weeks I was happy and in a good mood’ (*α* = 0.86, *N* = 2,882).

### Data Analysis

The data in this study were first prepared using IBM SPSS Statistics 27. The following analyses were performed using Mplus 7.3 (Muthén and Muthén, 1998–2012). LPAs were performed to look for homogenous subgroup patterns of study crafting behaviour (cf., Wang and Hanges, 2011). An LPA accounts for uncertainty about an individual’s true profile and, thus, an individual only contributes to the profile-specific means depending on their profile probability (Magidson and Vermunt, 2002). Since the assumption of the deterministic assignment of individuals to profiles is dropped, it is, therefore, also referred to as a probabilistic cluster analysis procedure (Bacher and Vermunt, 2010).

The profile analysis was performed according to the automatic BCH method (Asparouhov and Muthén, 2014; Bakk and Vermunt, 2015). As often recommended, the model was considered independently of its statistical relationship to covariates and criteria for this purpose (Vermunt, 2010; Asparouhov and Muthén, 2014; Bakk and Vermunt, 2015). In mixed distribution models, if the criteria are directly included in the calculation, the profiles may be determined not only by the original latent profile indicators, but also by the criteria and, thus, be biased or altered in number (Huang et al., 2010). All models (profile solutions) were computed with 1,000 initial value sets to reduce the risk of a local likelihood maximum, and each of these sets was processed with up to 500 iterations (Geiser, 2011).

The Akaike Information Criterion (AIC; Akaike, 1987), the Bayesian Information Criterion (BIC; Schwartz, 1978) and the sample-adjusted Bayesian Information Criterion (SABIC; Sclove, 1987) were used as information criteria to select the model with the most appropriate profile number. Lower values indicate a better model fit. In addition, the model-comparative Lo-Mendell-Rubin test (LMR; Lo et al., 2001; Asparouhov and Muthén, 2012) was used to select the model. The BIC and LMR tests together are recommended for choosing a model (Nylund et al., 2007), so these two were chosen as primary indicators. The entropy of the model was retained as an indicator of the quality of the classification (Celeux and Soromenho, 1996). The entropy measures the probability that any person from the sample is assigned to the best matching profile.

Based on Merians et al. (2019), the selected profile solution was validated using double cross-validation. For this purpose, the overall dataset was randomly split into two halves, and all profile solutions of the overall dataset were replicated with the first half. Using the previously described information criteria and the LMR test, the profile solutions were compared to exclude, as far as possible, a random distribution of the profile solutions based on the sample. Furthermore, the final profile solution from the first half, as well as the matching profile solution from the second half, were checked for their visual correspondence with the selected profile solution of the overall data set.

Following this, a criterion-related validity check of the latent profiles (Bacher and Vermunt, 2010)—the difference between the latent profiles in the criteria emotional exhaustion, study engagement, and well-being—was conducted. For this purpose, the output file of the final profile analysis of the total data set was used. In the automatic BCH method, the measurement model for the latent profile variable is specified during profile analysis, and the criteria are specified as such. The profile analysis is then computed independently of the criteria, but the output file displays the profile differences concerning the criteria. The results of the chi-square estimates and *p* values of each Wald test were used to test for differences between profiles in the outcome variables.

## Results

### Identifying Latent Study Crafting Profiles Among Students

Five latent profile models with different numbers of profiles ranging from two to six, were estimated. Regarding the choice of the best fitting profile number, Table 3 summarises the information criteria of AIC (Akaike, 1987), BIC (Schwartz, 1978), and SABIC (Sclove, 1987), as well as the entropy and the model-comparative LMR test (Nylund et al., 2007).

**Table 3:** Model comparison for different profile solutions.

*k*	AIC	BIC	SABIC	Entropy	LMR (*p*-value)
2	19,779.954	19,839.406	19,807.632	0.72	<0.001
3	19,354.315	19,437.548	19,393.065	0.71	<0.001
4	19,171.182	19,278.196	19,221.004	0.71	<0.001
5	19,050.546	19,181.340	19,111.439	0.66	<0.001
6	18,953.930	19,108.505	19,025.894	0.81	<0.097

*k*, number of latent profiles in the model; AIC, Akaike information criterion; BIC, Bayesian information criterion; SABIC, sample-adjusted BIC; LMR, Lo-Mendell-Rubin test for *k* vs. *k* − 1 profiles.

Among the possible profile solutions, AIC, BIC, and SABIC were first considered for the model fit. Although all information criteria decrease with increasing profiles, there is only minimal improvement above the 5-profile solution, favouring the 5-profile model. Additionally, the LMR test was conducted, comparing the respective *k*-profile model with the (*k* − 1)-profile model, with significant results indicating a better fit for the k-profile model (Nylund et al., 2007). In the present case, the values also favoured the 5-profile model because the 6-profile solution was not significantly better than the 5-profile solution, which, on the other hand, was better than the 4-profile solution.

We also report entropy, indicating that individuals are classified with more confidence (Van De Schoot et al., 2017). Entropy can take values from 0 to 1 and receives a maximum value of 1 when each participant perfectly fits to only one of the profiles, which would indicate that the latent profiles are completely discrete partitions (Van De Schoot et al., 2017). Following Greenbaum et al. (2005), the entropy should not be used to choose the number of profiles. Models with higher entropy should only be decisive for the decision if also supported by similar relative fit indexes (e.g. BIC; Ram and Grimm, 2009; Van De Schoot et al., 2017). In addition to the statistical fit of profile solutions, according to Uebersax (1994), model selection should be based on a model’s interpretability, parsimony, and meaningfulness. Even though the five-factor solution had a slightly lower entropy, as compared to solutions with less profiles, a further differentiation emerged (concerning average crafters, showing either more or less social crafting) compared to the four profiles. Therefore, we used the five profiles for further analyses.

**Figure 1:**
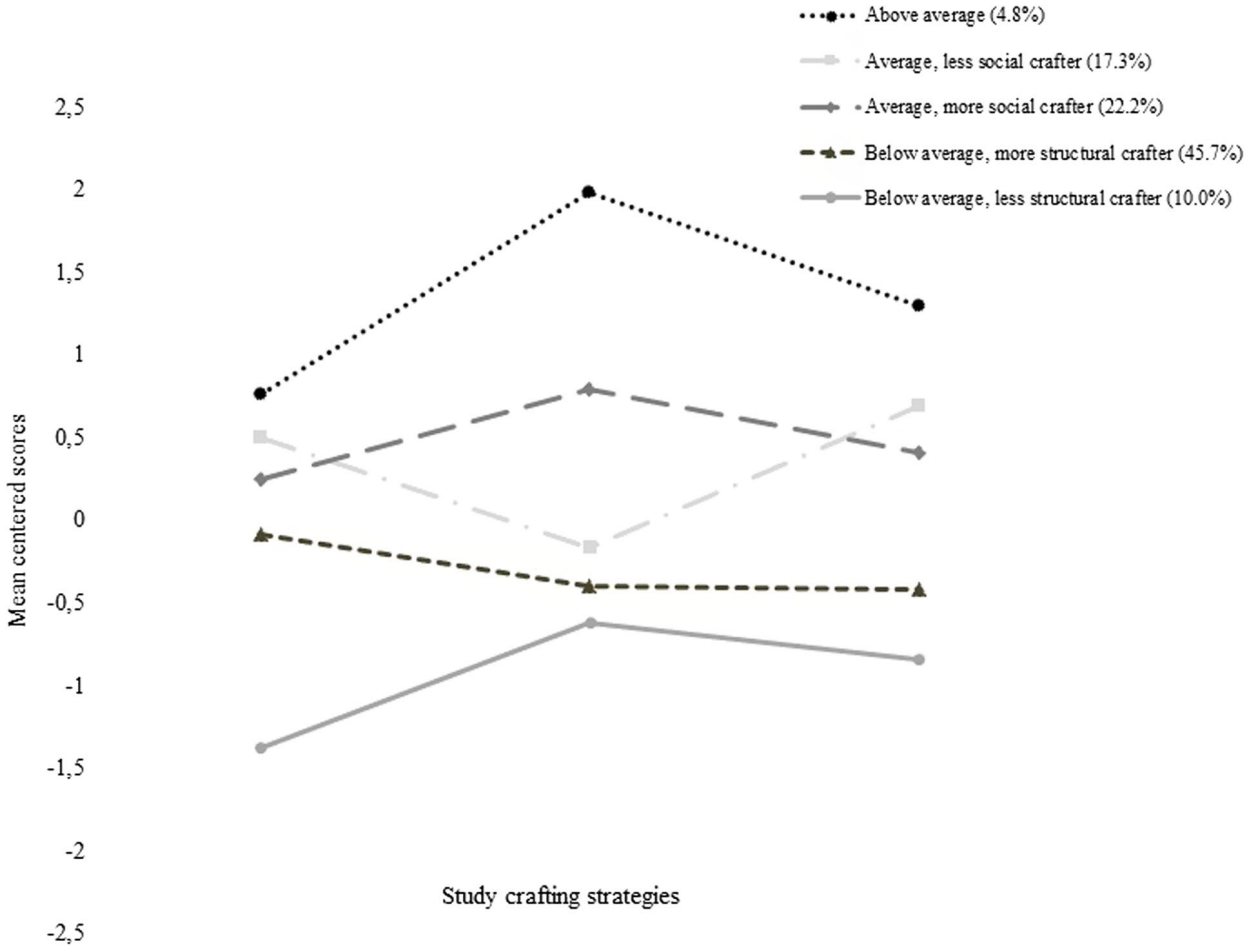
Mean centred scores on study crafting strategies for the five latent profiles.

Figure 1 shows the mean centered characteristic response behaviour of the five profiles in the final solution. The *above average crafting profile* was assigned to 4.8% of university students in our sample and is characterized by above average crafting in all three facets, with especially high values for social crafting. Two further profiles can be labeled as average crafters. These two profiles differ mainly in the usage of social crafting strategy. Hence we labeled these profiles as ‘*average crafting more social crafting*’ with a share of 22.2% and ‘*average crafting, less social crafting*’ with a share of 17.3%. Two further profiles show below average crafting in all three facets. These two profiles mainly differ in the usage of increasing structural resources, and were thus labeled ‘*below average, more structural crafting*’ represented by 45.7% of respondents, and ‘*below average, less structural crafting*,’ represented by 10.0% of our sample.

### Associations Between Study Crafting Profiles and Emotional Exhaustion, Engagement, and Well-Being

Table 4 shows the means, standard deviations, correlations, and Cronbach’s alphas of all used variables. The correlations show negative relationships between study crafting strategies and emotional exhaustion and positive relationships with study engagement and well-being.

**Table 4:** Study variables, means, standard deviations, correlations, and Cronbach’s alphas (*N* = 2,822).

Variables	Mean	SD	1	2	3	4	5	6
Increasing structural resources	3.97	0.77	(0.84)					
Increasing social resources	1.97	0.82	0.32	(0.71)				
Increasing challenging demands	2.40	0.89	0.41	0.44	(0.70)			
Emotional exhaustion	4.32	1.60	−0.13	−0.09	−0.11	(0.92)		
Study engagement	3.62	0.95	0.42	0.31	0.32	−0.37	(0.84)	
Well-being	11.99	5.23	0.23	0.09	0.16	−0.35	0.35	(0.99)

SD, standard deviation. Cronbach’s alphas are presented in parentheses in the diagonal. All correlations are significant at the *p* < 0.001 level.

**Figure 2:**
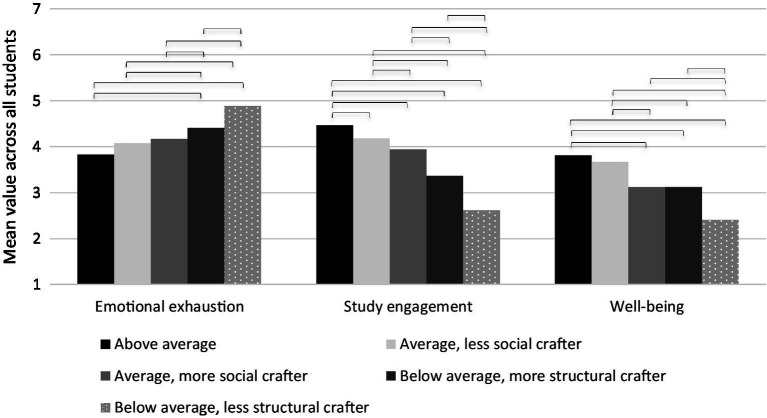
Mean values of outcomes for each profile. *N* = 2,822. Emotional exhaustion with values from 1 to 7. Study engagement and well-being with values from 1 to 6. Square brackets indicate significant differences according to Wald tests.

In order to test for the discriminant validity of the five profiles, we investigated differences in the concurrent assessment of emotional exhaustion, engagement, and well-being. Table 5 summarises the results from the automatic BCH approach (Asparouhov and Muthén, 2021) and reports chi-square estimates and *p* values of each Wald test to assess the equality of the profile-specific mean levels for each outcome (Bakk and Vermunt, 2015). Profile-specific mean levels for each outcome are illustrated in Figure 2.

**Table 5:** Wald tests (*N* = 2,822).

	Above average crafter		Average, less social crafter		Average, more social crafter		Below average crafter	
	*χ* ^2^	*p*	*χ* ^2^	*p*	*χ* ^2^	*p*	*χ* ^2^	*p*
*Emotional exhaustion*								
Average, less social crafter	1.38	0.241						
Average, more social crafter	3.02	0.082	0.34	0.560				
Below average	10.21	0.001	4.05	0.044	4.51	0.034		
Below average, less structural crafter	24.16	<0.001	19.93	<0.001	20.87	<0.001	9.61	0.002
*Study engagement*								
Average, less social crafter	5.63	0.018						
Average, more social crafter	19.89	<0.001	5.95	0.015				
Below average	111.57	<0.001	87.68	<0.001	93.78	<0.001		
Below average, less structural crafter	229.47	<0.001	247.79	<0.001	240.44	<0.001	77.48	<0.001
*Well-being*								
Average, less social crafter	0.57	0.450						
Average, more social crafter	11.24	<0.001	5.44	0.020				
Below average	23.50	<0.001	8.47	0.004	<0.01	0.970		
Below average, less structural crafter	34.68	<0.001	25.93	<0.001	8.26	0.004	8.72	0.003

As expected, inclusion in the profiles with high study crafting strategies (*above average crafters*) resulted in the lowest level of emotional exhaustion and the highest level of study engagement, as well as well-being. The *above average crafters* scored significantly higher in study engagement than any other group. Emotional exhaustion was only significantly lower in the *above average group* when compared to the two *below average crafter groups*. Regarding well-being significant differences were observed to three of the four other groups, with *average*, *less social crafter* being the exception. The *average crafter groups* showed significantly lower levels of emotional exhaustion and higher levels in study engagement, when compared to the two *below average crafter* groups. Regarding well-being only the *average, less social crafter group*, but not the *more social crafter group* scored higher than the *below average, more structural crafting group*. The two *below average crafting* groups showed significant differences in all three outcome variables, with the group *below average, more structural crafting* reporting lower emotional exhaustion, higher study engagement, and higher well-being.

Overall, this confirms the expected negative relationship between study crafting strategies and emotional exhaustion as proposed in Hypothesis 1a. The results are mainly consistent with the presumed positive influence of study crafting on study engagement and well-being, lending support to H1b and H1c.

## Discussion

The main contributions of this study are the application of the person-centered approach to the concept of study crafting, as well as the further differentiation of the approach (or active) crafting category, which has been suggested in the research of employee job crafting. We found five latent profiles distinguishing different patterns of study crafting dimensions. Our results provide a more differentiated pattern of distinct combinations of approach crafting than the study conducted by Mäkikangas (2018), who reported only two different profiles in an employee sample, which were labeled as passive and active crafting types. As our focus was on active or approach crafting strategies, we contributed to a refinement of this category. Respondents belonging to the five profiles could be categorized as above average, average, and below average crafters, with further differentiation for average crafters concerning more or less usage of social crafting strategies and for the below average crafters concerning more or less usage of increasing structural resources strategy. Within the group of above average crafters, we observed that they were especially pronounced concerning increasing social resources. The majority of students in our sample could be categorized as below average crafters with a share of 55.7% (45.7% below average, less structural crafting, and 10.0% below average, more structural crafting), next 39.5% of students were categorized as average crafters, including slightly more within the subgroup of using more social crafting strategies. Only a marginal share of students (4.8%) were classified as above average crafters.

Given the changes due to the COVID-19 pandemic, we can assume that increasing social resource is not easily possible in online classes. The mean scores of this dimension are lower than in the results of Mäkikangas (2018) job crafting profiles, and only 4.8% of our participants could be categorized as above average crafters, with especially high social crafting. A reason for this finding can be the social isolation due to online learning.

The five profiles showed discriminant validity in terms of emotional exhaustion, engagement, and general well-being. Altogether, the profiles were associated with health and well-being in the following descending order: *above average crafters* (1), *average, less social crafter* (2), *average, more social crafter* (3), *below average, more structural crafter* (4), *below average, less structural crafter* (5). Although profiles 1, 2, and 3 obviously show substantial differences in the level of crafting dimensions, these differences are not always noticeable in relation to the outcomes. Since profiles 1, 2, and 3 do not differ significantly for emotional exhaustion, this suggests that even a medium level of all crafting strategies seems favourable for students’ health and well-being. Profiles 4 and 5 differ significantly, indicating that students with low scores on all dimensions—mainly increasing structural resources—report exhaustion the most. Levels of study engagement, on the other hand, differ across the first three profiles and clearly suggest that the combination of crafting behaviour, as represented by (1) *above average crafting*, is most beneficial for engagement with its significant high expression of increasing social resources and high expression of increasing challenging demands. Again, the differences in the profiles of 4 and 5 are evident, meaning that students with low usage of crafting strategies show little engagement, which is consistent with previous findings (Rudolph et al., 2017; Lichtenthaler and Fischbach, 2019; Frederick and VanderWeele, 2020; Hu et al., 2020). Concerning well-being, profile 1 (*above average crafter*) and 2 (*average, less social crafter*) do not differ, but profile 1 (*above average crafter*) differs from 3 (*average, more social crafter*), and profile 2 (*average, less social crafter*) differs from 3 (*average, more social crafter*). Surprisingly, the students in profile 2—characterized by average values of crafting—report significantly higher scores for well-being, even though they are less likely to use the increasing social crafting strategy than in profile 3. These results are inconsistent with previous findings from the occupational context (Boehnlein and Baum, 2020).

### Theoretical Implications

This study provides further evidence (e.g., Dormann and Guthier, 2019; Ferreira, 2020; Körner et al., 2021) that the job crafting concept can be adapted successfully to the higher education context. Study crafting, in general, showed comparably positive associations with engagement and general well-being, as well as a negative association with emotional exhaustion, as has been reported for employees (Rudolph et al., 2017; Lichtenthaler and Fischbach, 2019; Frederick and VanderWeele, 2020; Hu et al., 2020). In addition, the presented results align with previous studies on study crafting (Ferreira, 2020; Körner et al., 2021).

Our study suggests that the concept of approach or active crafting can further be differentiated. However, the five clusters in our study differ mainly with regard to the overall level of the three selected crafting dimensions. Increasing social resources turned out to be the driver for a more fine-grained differentiation among students using crafting strategies on an average level. Surprisingly, profile 2 (*average, less social crafter*), with a lower increase in social resources, combined with average other crafting strategies, showed higher study engagement compared to profile 3 (*average, more social crafter*), which is characterized by higher values in increasing social resources. Combining an average increase in structural resources and an average increase in challenging demands seems to be better for engagement when combined with a lower increase in social resources. This might indicate that students who already have sufficient social resources and thus have a lower need in further increase of social resources might be better off. This finding provides a more fine-grained picture compared to previous research on job crafting in variable-centered studies, where higher crafting behaviour in all dimensions has been consistently related to higher engagement (Rudolph et al., 2017; Lichtenthaler and Fischbach, 2019). For the interpretation of these results, the cross-sectional study design needs to be considered as a limitation. In addition, we do not know the individual reasons why some students engage in more or less certain crafting strategies. One potential reason to engage less in gaining a certain resource can be that this resource is already sufficiently available. Future studies might thus consider the motivation to engage in crafting behaviour, as well as an evaluation of the current status regarding structural and social resources as well as challenging demands.

A similar pattern emerged for well-being. Again, profile 2 (*average, less social crafter*) was associated with significantly higher well-being than profile 3 (*average, more social crafter*). Profiles 2 (*average, less social crafter*) and 3 (*average, more social crafter*) did not differ concerning emotional exhaustion, and no significant differences between profiles 1 (*average, less social crafter*) and profile 3 (*average, more social crafter*) were observed, despite the fact that profile 1 (*above average crafter*) is characterized by overall higher values in all study crafting dimensions. From these results, it can be concluded that, at least in the study context, it is not only the overall extent of crafting behaviour that is essential for health and well-being. Even though increasing social resources in isolation showed a significant negative correlation with emotional exhaustion and significant positive correlations with engagement and well-being, these effects were not observable when combined with increasing structural resources and increasing challenge demands.

### Limitations and Future Research

This research is a cross-sectional investigation of study crafting and emotional exhaustion, work engagement, and well-being. Consequently, we cannot draw any causal conclusions. Whether study crafting leads to more health and well-being or health and well-being lead to more or less study crafting remains an open question that should be addressed in studies using multiple waves of data and cross-lagged panel analyses.

This study offers some insight into person-centered study crafting dimensions, despite its exploratory nature. Even though we used data from a considerably large sample, we cannot be sure that other samples will not show different combinations of study crafting behaviour. Even though the entropy should not be the reason to choose the number of profiles (Greenbaum et al., 2005), the entropy value of the five profile solution was the lowest (0.66), which indicates that some other profile solutions (e.g., four profiles) might provide a slightly more reliable categorization of students. However, the five profile solution provided a further differentiation, which proved to show incremental validity. Further research should examine whether the same or similar profiles are found in other student samples. Particular attention should also be paid to examining students in other parts of the world, including other European samples at large universities, as study conditions vary considerably internationally.

It should be noted that the study crafting items were not yet validated and that the short form of the job crafting scale by Tims et al. (2012) was adapted. We want to highlight the new combination and adaption of existing items. The items about increasing social resources included the support and feedback of lecturers and not fellow students. Therefore, the contradictory results of increasing social resources should be considered cautiously.

When interpreting the data, it should be noted that the survey took place during the summer of 2020, with study conditions characterized by online teaching due to the pandemic situation. It seems plausible that these circumstances impacted opportunities to engage in study crafting.

### Practical Implications

The findings of this study have several important implications for practice. Since study crafting has been shown to be positively related to engagement and general well-being, as well as negatively related to emotional exhaustion, it seems warranted to search for ways to train students in study crafting. To the best of our knowledge, no study crafting interventions have been reported in research to date. Preventive interventions focussing on the reduction of stress in students mainly focused on stress regulation techniques such as mindfulness (Voss et al., 2020; Světlák et al., 2021). One meta-analysis reports that job crafting is trainable overall, but not all dimensions seem to be equally malleable. According to Oprea et al. (2019), overall structural and social resources increases are not reported.

However, according to our results in the study context, it is essential to increase structural resources, which should also be promoted in combination with increasing challenging demands. Moreover, we cannot make any recommendation regarding the reducing hindering demands dimension in studies because it was not considered. However, in interventions, students could reflect on their personal values, strengths, and passions in a person and task analysis. In addition, the content of a study crafting training could include sharing information about the study crafting dimensions and then setting SMART goals. SMART is an acronym that stands for Specific—Measurable—Achievable—Realistic—Timely. For instance, a goal in increasing structural resources could be to learn new methodological development outside of the regular curriculum; resources are gained, and study-related self-efficacy may grow. Students could keep a checklist to solidify their goals (Gordon et al., 2018).

## Conclusion

This study was designed to determine study crafting latent profiles and their association to emotional exhaustion, study engagement, and general well-being. Considered together, the results suggest that the combination of the study crafting dimensions of increasing structural resources and increasing challenging demands seem to be especially beneficial. Specifically, a medium use of strategies is associated with better general well-being. In addition to medium usage of increasing social resources, the combination of higher usage of increasing structural resources and high increasing challenging demands is associated with greater engagement and well-being among students. This approach will prove helpful in expanding our understanding of how study crafting strategies are used and combined. The reported results imply that interventions in study crafting show promise for promoting students’ health and well-being.

## Data Availability Statement

The raw data supporting the conclusions of this article will be made available by the authors, without undue reservation.

## Ethics Statement

The studies involving human participants were reviewed and approved by Johannes Gutenberg University‘s Psychology Research Ethics Committee (2020-JGU-psychEK-S008). The patients/participants provided their written informed consent to participate in this study.

## Author Contributions

All authors were involved in developing the research materials, design, and data collection. SS, LM, and TR analysed the data, data interpretation, and the formation of guiding principles. SS wrote most parts of the methods and results. LM and TR wrote the introduction and discussion. AW, JR, SH, AT, MS, PD, MB, BS, and PS reviewed this manuscript. All authors contributed to the article and approved the submitted version.

## Funding

The Healthy Campus Mainz project is funded by BARMER health insurance and carried out with the support of the Johannes Gutenberg University of Mainz and the University Medical Centre of the Johannes Gutenberg University of Mainz.

## Conflict of Interest

The authors declare that the research was conducted in the absence of any commercial or financial relationships that could be construed as a potential conflict of interest.

## Publisher’s Note

All claims expressed in this article are solely those of the authors and do not necessarily represent those of their affiliated organizations, or those of the publisher, the editors and the reviewers. Any product that may be evaluated in this article, or claim that may be made by its manufacturer, is not guaranteed or endorsed by the publisher.
